# Safety and immune cell kinetics after donor natural killer cell infusion following haploidentical stem cell transplantation in children with recurrent neuroblastoma

**DOI:** 10.1371/journal.pone.0225998

**Published:** 2019-12-13

**Authors:** Young Bae Choi, Meong Hi Son, Hee Won Cho, Youngeun Ma, Ji Won Lee, Eun-Suk Kang, Keon Hee Yoo, Jung Hyun Her, Okjae Lim, Miyoung Jung, Yu Kyeong Hwang, Ki Woong Sung, Hong Hoe Koo

**Affiliations:** 1 Department of Pediatrics, Chungbuk National University Hospital, Cheongju, Republic of Korea; 2 Department of Pediatrics, Samsung Medical Center, Sungkyunkwan University School of Medicine, Seoul, Republic of Korea; 3 Department of Pediatrics, Seoul National University Bundang Hospital, Sungnam, Republic of Korea; 4 Department of Laboratory Medicine and Genetics, Samsung Medical Center, Sungkyunkwan University School of Medicine, Seoul, Republic of Korea; 5 Cell Therapy Research Center, GC LabCell, Yongin, Republic of Korea; 6 MOGAM Institute for Biomedical Research, Yongin, Republic of Korea; European Institute of Oncology, ITALY

## Abstract

**Introduction:**

Under the hypothesis that early natural killer cell infusion (NKI) following haploidentical stem cell transplantation (haplo-SCT) will reduce relapse in the early post-transplant period, we conducted a pilot study to evaluate the safety and feasibility of NKI following haplo-SCT in children with recurrent neuroblastoma who failed previous tandem high-dose chemotherapy and autologous SCT.

**Methods:**

We used the high-dose ^131^I-metaiodobenzylguanidine and cyclophosphamide/fludarabine/anti-thymocyte globulin regimen for conditioning and infused 3 × 10^7^/kg of *ex-vivo* expanded NK cells derived from a haploidentical parent donor on days 2, 9, and 16 post-transplant. Interleukin-2 was administered (1 × 10^6^ IU/m^2^/day) subcutaneously to activate infused donor NK cells on days 2, 4, 6, 9, 11, 13, 16, 18, and 20 post-transplant.

**Results:**

Seven children received a total of 19 NKIs, and NKI-related acute toxicities were fever (n = 4) followed by chills (n = 3) and hypertension (n = 3); all toxicities were tolerable. Grade ≥II acute GVHD and chronic GVHD developed in two and five patients, respectively. Higher amount of NK cell population was detected in peripheral blood until 60 days post-transplant than that in the reference cohort. Cytomegalovirus and BK virus reactivation occurred in all patients and Epstein-Barr virus in six patients. Six patients died of relapse/progression (n = 5) or treatment-related mortality (n = 1), and one patient remained alive.

**Conclusion:**

NKI following haplo-SCT was relatively safe and feasible in patients with recurrent neuroblastoma. Further studies to enhance the graft-versus-tumor effect without increasing GVHD are needed.

## Introduction

The development of high-dose chemotherapy and autologous stem cell transplantation (HDCT/auto-SCT) has improved treatment outcomes of patients with high-risk neuroblastoma in recent decades [1–4]. However, many patients with high-risk neuroblastoma experience relapse after HDCT/auto-SCT, and in these patients, allogeneic SCT (allo-SCT) with graft-versus-tumor (GVT) effects might be a treatment option [4]. Recently, haploidentical SCT (haplo-SCT) with or without high-dose ^131^I-metaiodobenzylguanidine (HD-MIBG) treatment has been performed as an attempt to increase the anti-tumor effect for patients with recurrent neuroblastoma and showed tolerable toxicity and potential anti-tumor effects [5,6].

In haplo-SCT in which T cells are usually depleted to prevent unacceptable graft-versus-host disease (GVHD), donor natural killer (NK) cells may play an important role in eliminating residual tumor cells until T cell recovery [7]. NK cells are innate effector lymphocytes and have cytotoxicity against tumor cells with decreased expression of major histocompatibility class I antigen [8,9]. The activity of NK cells is controlled by networking of activating and inhibitory receptors [10]. Previous studies have shown that selection of donors with killer cell immunoglobulin-like receptors (KIR) mismatched with recipient HLA or group B KIR haplotype improved transplant outcomes in several malignancies [11–15]. Neuroblastoma cells have been reported to have decreased class I HLA expression, which suggests that NK cell therapy may be effective in killing neuroblastoma cells [16]. Our previous study showed that KIR/HLA-ligand mismatched haplo-SCT might improve outcomes in children with recurrent neuroblastoma; however, most relapse/progression occurred in the early post-transplant period, suggesting the need for further effective treatment to prevent early relapse after haplo-SCT [17].

Clinical trials exploring the feasibility of donor-derived NK cell infusion (NKI) after haplo-SCT have been performed in patients with several malignancies [18–21]. Although clinical trials using NKI for recurrent neuroblastoma have been reported recently [22,23], studies on NKI after haplo-SCT in children with neuroblastoma are limited [24]. Thus, under the hypothesis that donor NKI after haplo-SCT may be helpful in preventing early relapse and improving survival, we performed a pilot study to explore the safety and feasibility of NKI following haplo-SCT in children with recurrent neuroblastoma who failed tandem HDCT/auto-SCT.

## Materials and methods

### Ethics statement

This study was approved by the Institutional Review Board of Samsung Medical Center and The Korean Food and Drug Administration and is registered at ClinicalTrials.gov with the registration number #NCT01807468. All parents gave written informed consent before enrollment. Patient records/information were anonymized and de-identified prior to analysis.

### Patients

Patients with neuroblastoma who experienced relapse/progression after tandem HDCT/auto-SCT from January 2012 to December 2014 without major organ dysfunction were eligible for this study.

### Treatment prior to haplo-SCT

Salvage chemotherapy was administered in order to reduce the tumor burden as much as possible prior to haplo-SCT. An ICE (ifosfamide + carboplatin + etoposide) regimen was used for first-line salvage treatment, and a TC (topotecan + cyclophosphamide) regimen was used for second-line salvage chemotherapy in patients with severe bone marrow suppression or refractory response with the first-line regimen. The duration of salvage chemotherapy prior to haplo-SCT depended on tumor response and patient tolerance. Tumors were surgically resected whenever possible. Local radiotherapy was also delivered to recurrent or metastatic sites whenever possible.

### Donor selection

Typing of HLA A, B, C, DRB1, and DQB1 was performed using high-resolution PCR sequence-based typing, and KIR genotyping was performed from donor DNA samples using a PCR-based sequence-specific oligonucleotide technique. A KIR/HLA-ligand mismatch was defined by incompatibility between the inhibitory donor KIR and recipient HLA class I alleles, as previously described [25]. Donor KIR haplotypes were categorized as AA (homozygous for group A KIR haplotypes) or BX [either one (A/B heterozygotes) or two (B/B homozygotes) group B haplotypes]. The KIR B haplotype-defining loci were *KIR2DL5*, *2DS1*, *2DS2*, *2DS3*, *2DS5*, or *3DS1* [11]. Genotypes were also assigned for the centromeric and telomeric regions of the KIR locus. A haploidentical parent donor with KIR/HLA-ligand mismatch and/or KIR BX haplotype was preferred.

### NK cell generation and stem cell collection

For NK cell production, haploidentical parent donors underwent lymphapheresis on day -28, and CD3^+^ cell–depleted peripheral blood mononuclear cells (PBMCs) were frozen at -196°C. Peripheral blood mononuclear cells were thawed (days -12, -5, and 2) 14 days before each of the three planned infusions (days 2, 9, and 16) to allow each preparation and infusion of fresh cells. The thawed PBMCs expanded as described previously under good manufacturing practice conditions [26]. Briefly, CD3^+^ cell–depleted PBMCs were expanded at a seeding concentration of 2 × 10^5^ cells/mL in CellGro SCGM serum-free medium (CellGenix, Germany) with 1% autologous plasma, 1 × 10^6^ cells/mL irradiated (2,000 rad) autologous PBMCs, 10 ng/mL anti-CD3 monoclonal antibody (Orthoclon, Switzerland), and 500 IU/mL of interleukin-2 (IL-2; Proleukin, Switzerland) in an A-350N culture bag (NIPRO, Japan). NK cells were fed fresh medium with 500 IU/mL of IL-2 every 2 days until they were harvested after 14 days. The cytotoxicity of *ex-vivo* expanded donor NK cells was measured using K562, SK-N-SH, and NB-1691 cells by calcein releasing assay. For peripheral blood stem cell (PBSC) collection, haploidentical parent donors received 5–10 μg/kg of G-CSF subcutaneously once daily for four days; PBSCs were collected and transplanted without manipulation on day 0.

### Conditioning

At 21 days prior to transplant, all children received a single 1-hour intravenous infusion of ^131^I-MIBG (18 mCi/kg) with potassium iodide for thyroid protection and intravenous hydration. A cyclophosphamide (cyclophosphamide 60 mg/kg on days -7 and -6) + fludarabine (30 mg/m^2^ on days -5 to -1) + rabbit anti-thymocyte globulin (Thymoglobulin, Genzyme; 2.5 mg/kg on days -4 to -1) regimen was used for conditioning.

### NKI

Patients received 3 × 10^7^/kg of *ex-vivo* expanded donor NK cells on days 2, 9, and 16 post-transplant. Donor NK cells were infused over 1 hour through a central venous catheter after pheniramine pre-treatment. Patients received IL-2 (1 × 10^6^ IU/m^2^/day) subcutaneously to activate infused donor NK cells on days 2, 4, 6, 9, 11, 13, 16, 18, and 20. On the day of NKI, IL-2 was administered after a 4-hour observation period post-NKI.

### GVHD prophylaxis and treatment

Cyclosporine (CSA) and short-course methotrexate were used to prevent GVHD. CSA was administered from day -1 at a dose adjusted to maintain blood concentration in the range of 150–300 ng/mL. Methotrexate was administered at a dose of 15 mg/m^2^ on day 1 and at 10 mg/m^2^ on days 3 and 6, followed by folic acid rescue. The timing and speed of CSA tapering were determined by GVHD and tumor status of each patient. If the patient did not achieve complete response (CR), early tapering of CSA was considered to enhance GVT. If acute GVHD developed during CSA prophylaxis or tapering, the CSA dose was increased. If ≥ grade II acute GVHD continued despite an increase in CSA dose, methylprednisolone (1–2 mg/kg/day) was added with subsequent tapering in responsive cases. In refractory GVHD, mycophenolate mofetil was added to reduce use of steroid. Acute and chronic GVHD were assigned grades and stages based on previously described standard clinical criteria [27].

### Infection surveillance and prophylaxis

Antifungal prophylaxis was administered until hospital discharge or during steroid treatment. Acyclovir was used to prevent viral reactivation by day 30, and trimethoprim-sulfamethoxazole was used from engraftment to day 180 or until immunosuppressant discontinuation. Cytomegalovirus (CMV), Epstein-Barr virus (EBV), and BK virus (BKV) surveillance were performed weekly during the first three months post-transplant and then monthly thereafter if no viral reactivation occurred. If CMV or EBV load was increasing, ganciclovir or rituximab was started as preemptive therapy, respectively.

### Chimerism study and immune monitoring

Donor/recipient chimerism was evaluated at 30, 60, 90, and 180 days post-transplant in peripheral blood. Immunologic recovery was assessed by immunophenotyping of PBMCs (CD3^+^, CD19^+^, and CD16^+^CD56^+^CD3^–^ cells) from recipients at 16, 30, 60, 90, 180, and 270 days post-transplant. In three patients (patient #4, #5, and #7), granulocyte-derived myeloid-derived suppressor cells (MDSCs) by lymphogating of Lin^–^CD14^–^HLA-DR^–^CD11b^+^CD33^+^CD15^+^ cells were analyzed to identify the association between the levels of these immune cells and relapse/progression [28].

### Toxicity and response assessment

NKI-related immediate adverse reactions were defined as adverse reactions that developed from initiation of NKI to 4 hours after completion of NKI. Toxicity was recorded according to the common toxicity criteria (version 4.0) outlined by the US National Cancer Institute. Tumor response evaluation was performed prior to HD-MIBG treatment and every three months for the first year post-transplant. International response criteria for neuroblastoma were used to evaluate treatment response [29].

### Statistical analysis

To serve as a reference cohort, we identified seven patients who experienced recurrent/progressive neuroblastoma between March 2012 and October 2014 from our previously reported cohort who underwent HD-MIBG treatment and haplo-SCT without NKI in our hospital [17]. Briefly, the reference cohort received ICE or TC regimens (± local radiotherapy) to reduce the tumor burden prior to haplo-SCT. Further ^131^I-MIBG (18 mCi/kg) was administered prior to reduced-intensity conditioning (cyclophosphamide + fludarabine + rabbit anti-thymocyte globulin) without NKI. Six of the 7 patients in the reference cohort experienced acute GVHD (grade I in five and grade III in one), and four patients experienced chronic GVHD (two mild and two severe). The differences in immune reconstitution after haplo-SCT were analyzed between the cohort in this study and the reference cohort using repeated measures ANOVA and Mann–Whitney test. Relapse/progression-free survival was calculated using Kaplan–Meier method and comparisons between survival curves were performed using the log-rank test. The results with a *P* value of < 0.05 were considered significant.

## Results

### Patients

Seven patients with recurrent neuroblastoma underwent a total of 19 NKIs after haplo-SCT; six patients completed 3 scheduled NKIs, and one patient (patient #6) received only the first NKI on day 2 due to failure of NK cell production thereafter. Patient characteristics prior to haplo-SCT are listed in Table 1. Patients received 4–7 cycles of salvage chemotherapy prior to haplo-SCT. Two patients underwent surgery, and four patients received local radiotherapy. Tumor status at haplo-SCT was CR in one patient, very good partial response in two, and partial response in four.

**Table 1:** Patient characteristics.

Patient #	Age (y) at Dx.	Stage at Dx	*MYCN* status	HDCT1 regimen	HDCT2 regimen	Interval (m) to relapse^a^	Age (y) at relapse	Relapsed sites	Treatment prior to haplo-SCT	Tumor status at haplo-SCT
**1**	3.3	4	NA	CEC	MIBG-TM	16	5.6	LNs	CT×4, L-RT	PR
**2**	3.5	4	A	CEC	MIBG-TM	32	7.2	Bone, BM	CT×6	VGPR
**3**	1.5	4	A	TTC	MEC	75	8.6	Bone, BM, brain	CT×5	PR
**4**	2.4	4	NA	CEC	MIBG-TM	12	4.4	Primary, LNs	Surgery, CT×6, L-RT	CR
**5**	3.1	4	NA	CEC	MIBG-TM	12	5.2	Brain, bone	Surgery, CT×7, L-RT	VGPR
**6**	3.3	4	A	CEC	MIBG-TM	45	5.9	Bone, BM, brain	CT×6	PR
**7**	1.5	4	NA	CEC	MIBG-TM	19	4.1	LNs	CT×5, L-RT	PR

Dx, diagnosis; NA, not amplified; A, amplified; HDCT1, first high-dose chemotherapy; HDCT2, second HDCT; RIST, reduced intensity stem cell transplantation; CEC, carboplatin + etoposide + cyclophosphamide; MIBG-TM, high-dose ^131^I-metaiodobenzylguanidine treatment + thiotepa + melphalan; MEC, melphalan + carboplatin + etoposide; LN, lymph node; BM, bone marrow; LMS, leptomeningeal seeding; CT, chemotherapy; L-RT, local radiotherapy; PR, partial response; MR, mixed response; VGPR, very good PR.

^a^Interval between HDCT2 and relapse/progression.

### Graft composition

Graft information is shown in Table 2. Six haploidentical donors had at least one KIR/HLA-ligand mismatch, and five donors had BX haplotype. A median of 22.7 × 10^8^ (range, 16.8–35.3) total nucleated cells/kg including medians of 13.1 (range, 6.5–30.1) × 10^6^ CD34^+^ cells/kg and 5.6 (range, 2.1–6.5) × 10^8^ CD3^+^ cells/kg were transplanted.

**Table 2:** Graft information, engraftment, and chimerism.

**Patient #**	**Donor relation**	**HLA match**	**KIR/HLA-ligand mismatch**	**Donor KIR haplotype (Cen/Tel)**	**No. of cells transplanted**			**Engraftment (day)**		**Donor chimerism (%)**		
					TNC (10^8^/kg)	CD34^+^ (10^6^/kg)	CD3^+^ (10^8^/kg)	ANC 500/μL	PLT 20,000/μL	Day 30	Day 60	Day 90
**1**	Mother	9/10	None	A/A, A/A	35.3	11.0	5.6	12	18	100	100	100
**2**	Mother	5/10	2DL1^a^, 3DL2^a^	A/A, A/B	17.1	9.2	4.8	13	27	100	100	100
**3**	Father	5/10	2DL1	A/A, A/A	16.8	13.1	2.6	11	16	99.8	99.8	99.2
**4**	Father	6/10	2DL1, 3DL2	A/A, A/B	32.3	30.1	5.8	11	19	100	100	100
**5**	Father	5/10	2DL1^a^, 3DL1, 3DL2	A/A, A/B	16.8	13.6	2.1	12	20	100	100	100
**6**	Mother	6/10	2DL1, 3DL2^a^	A/B, A/A	28.0	6.5	6.5	13	14	100	100	100
**7**	Mother	5/10	2DL1^a^, 3DL2^a^	A/B, A/A	22.7	15.7	6.5	12	17	100	99.1	100

HLA, human leukocyte antigen; KIR, killer cell immunoglobulin-like receptor; Cen, centromere; Tel, telomere; TNC, total nucleated cells; ANC, absolute neutrophil count; PLT, platelet count.

^a^Unlicensed KIR/HLA-ligand mismatch between donor and recipient.

### Characterization of *ex vivo*-expanded NK cells

**Fig 1:**
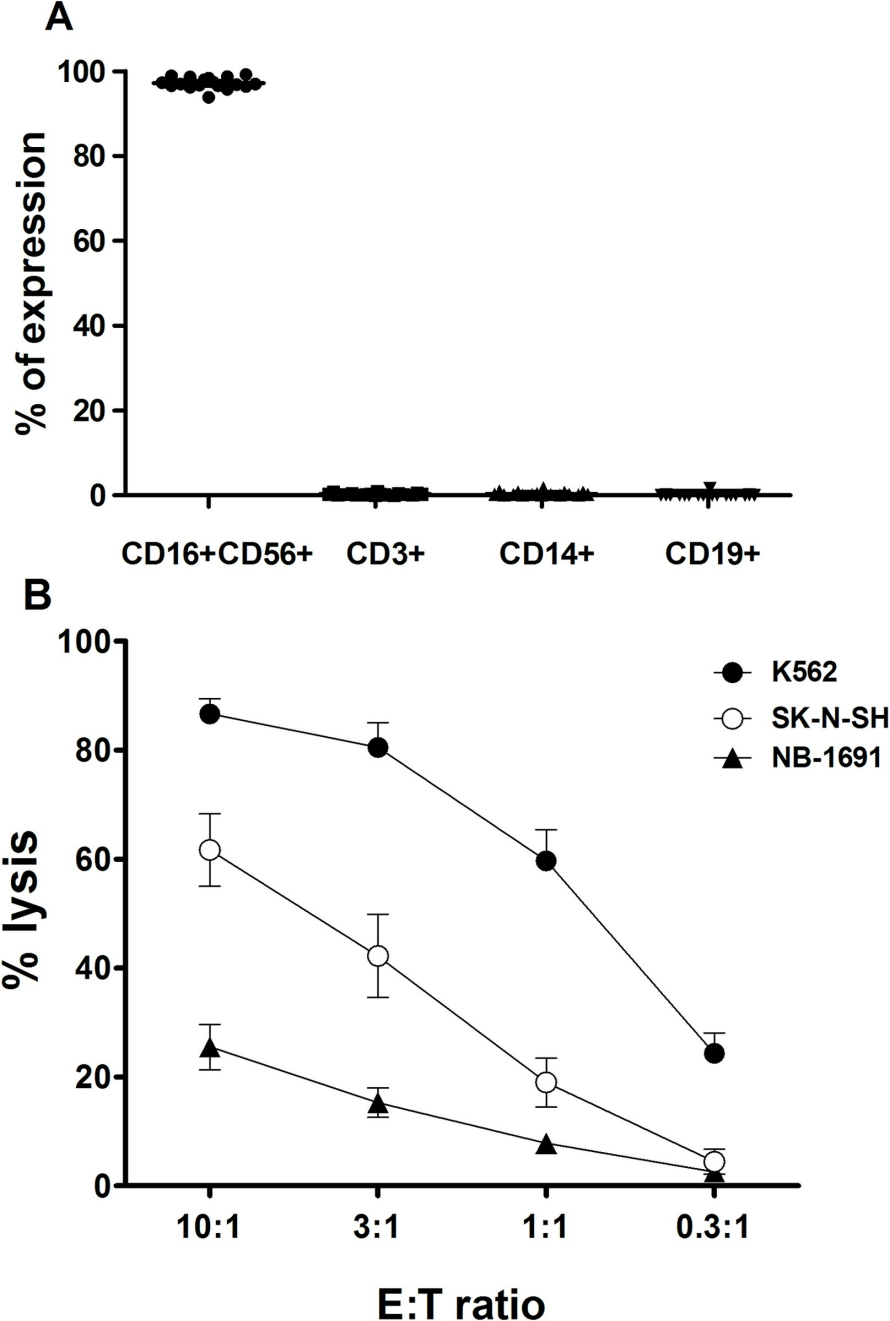
Characterization of *ex vivo*-expanded NK cells. (A) The percentages of CD16^+^CD56^+^, CD3^+^, CD14^+^, and CD19^+^ cells were analyzed by flow cytometric analyses. (B) Cytotoxicity of expanded NK cells against the K562, SK-N-SH, and NB-1691 cell line was analyzed by calcein releasing assay with the indicated E:T ratio. Each point represents mean ± SD.

NK cells were composed of enriched CD16^+^CD56^+^ cells (97.18 ± 1.33%) with minimal contamination of CD3^+^ cells (0.35 ± 0.25%), CD14^+^ cells (0.45 ± 0.49%), and CD19^+^ cells (0.10 ± 0.40%; Fig 1A). In a cytotoxicity assay, NK cells showed potent cytolytic activity against K562 cells, SK-N-SH cells, and NB-1691 cells (Fig 1B).

### NKI-related immediate adverse reactions

NKI-related immediate adverse reactions observed during or after NKI are summarized in Table 3. Out of 19 NKIs in seven patients, fever (n = 4) was most the common adverse reaction, followed by chills (n = 3) and hypertension (n = 3); however, these adverse reactions were manageable and transient. One patient (patient #3) experienced grade 3 hypertension after NKI, which disappeared after anti-hypertensive treatment. The only adverse reaction related to IL-2 treatment was fever, which occurred in all patients.

**Table 3:** NKI-related immediate toxicity profiles in 19 NKIs.

Toxicities	Grade 1–2	Grade 3–4	Total
**Fever**	4 (21.1%)	0	4 (21.1%)
**Chills**	3 (15.8%)	0	3 (15.8%)
**Hypertension**	2 (10.5%)	1 (5.3%)	3 (15.8%)

NKI, natural killer cell infusion.

### Regimen-related short-term toxicities

There were no short-term toxicities related to HD-MIBG treatment. After reduced-intensity conditioning, neutropenic fever (n = 7), hypokalemia (n = 6), elevated liver enzymes without veno-occlusive disease (n = 3), and diarrhea (n = 1) were common conditioning regimen-related grade ≥ 3 toxicities. However, these toxicities were manageable, and there was no regimen-related death.

### Hematologic recovery and chimerism

The median times required to reach an absolute neutrophil count of 500/μL and a platelet count of 20,000/μL without transfusion for 7 days were 12 (range, 11–13) days and 18 (range, 14–27) days, respectively (Table 2). Complete donor chimerism (> 99%) was achieved at day 30 in all patients and was maintained thereafter.

### GVHD

Acute GVHD developed in all patients (grade I in five and grade II in two), and chronic GVHD developed in five patients (mild in two, moderate in two, and severe in one; Table 4). CSA was tapered before 2 months post-transplant in four patients who showed PR or VGPR to enhance GVT effects, of which two patients (patients #3 and #5) showed no chronic GVHD and the remaining two patients (patients #6 and #7) showed mild and moderate chronic GVHD, respectively.

**Table 4:** GVHD and final outcome.

Patient #	Acute GVHD				Onset of acute GVHD (d)	Onset of CSA tapering (d)	Chronic GVHD^a^	Onset of chronic GVHD (d)	First time of discontinuation of CSA (m)	Tumor status at haplo-SCT	Tumor status at day 90	Final outcome (Follow-up from transplant)
	Skin	Gut	Liver	Overall								
**1**	1	0	0	I	22	194	Severe	56	–	PR	PR	DOD at 16 m, PD at 9 m
**2**	2	0	0	I	13	100	Mild	138	–	VGPR	CR	DOD at 23 m, relapse at 9 m
**3**	2	0	0	I	6	51	None	–	22	PR	CR	DOD at 29 m, relapse at 9 m
**4**	3	1	0	II	11	131	Moderate	103	15	CR	CR	DOD at 16 m, relapse at 6 m
**5**	2	0	0	I	5	47	None	–	15	VGPR	VGPR	Alive at 45 m in CR, PD at 6 m
**6**	2	0	0	I	1	54	Mild	42	7	PR	PD	DOD at 8 m in PD, PD at 2 m
**7**	3	0	0	II	3	33	Moderate	180	4	PR	PR	TRM in PR at 10 m

GVHD, graft-versus-host disease; CSA, cyclosporine; haplo-SCT, haploidentical stem cell transplantation; PR, partial response; DOD, died of disease; VGPR, very good PR; CR, complete response; PD, progressive disease; TRM, treatment-related mortality.

^a^Chronic GVHD was graded according to the National Institutes of Health consensus criteria.

### Infectious complications

Bloodstream bacterial infection developed in two patients. No patient developed invasive fungal infection. All seven patients experienced CMV reactivation and received preemptive ganciclovir treatment, and no patient experienced CMV disease. EBV reactivation was observed in six patients, four of whom received preemptive treatment with rituximab. One patient (patient #5) developed post-transplant lymphoproliferative disease, which improved after rituximab treatment. BKV reactivation was observed in all seven patients, and two patients (patient #2 and #6) experienced BKV-associated hemorrhagic cystitis. A patient (patient #7) with moderate chronic GVHD died from *Pneumocystis jirovecii* pneumonia at 10 months post-transplant without tumor progression.

### Immune monitoring

**Fig 2:**
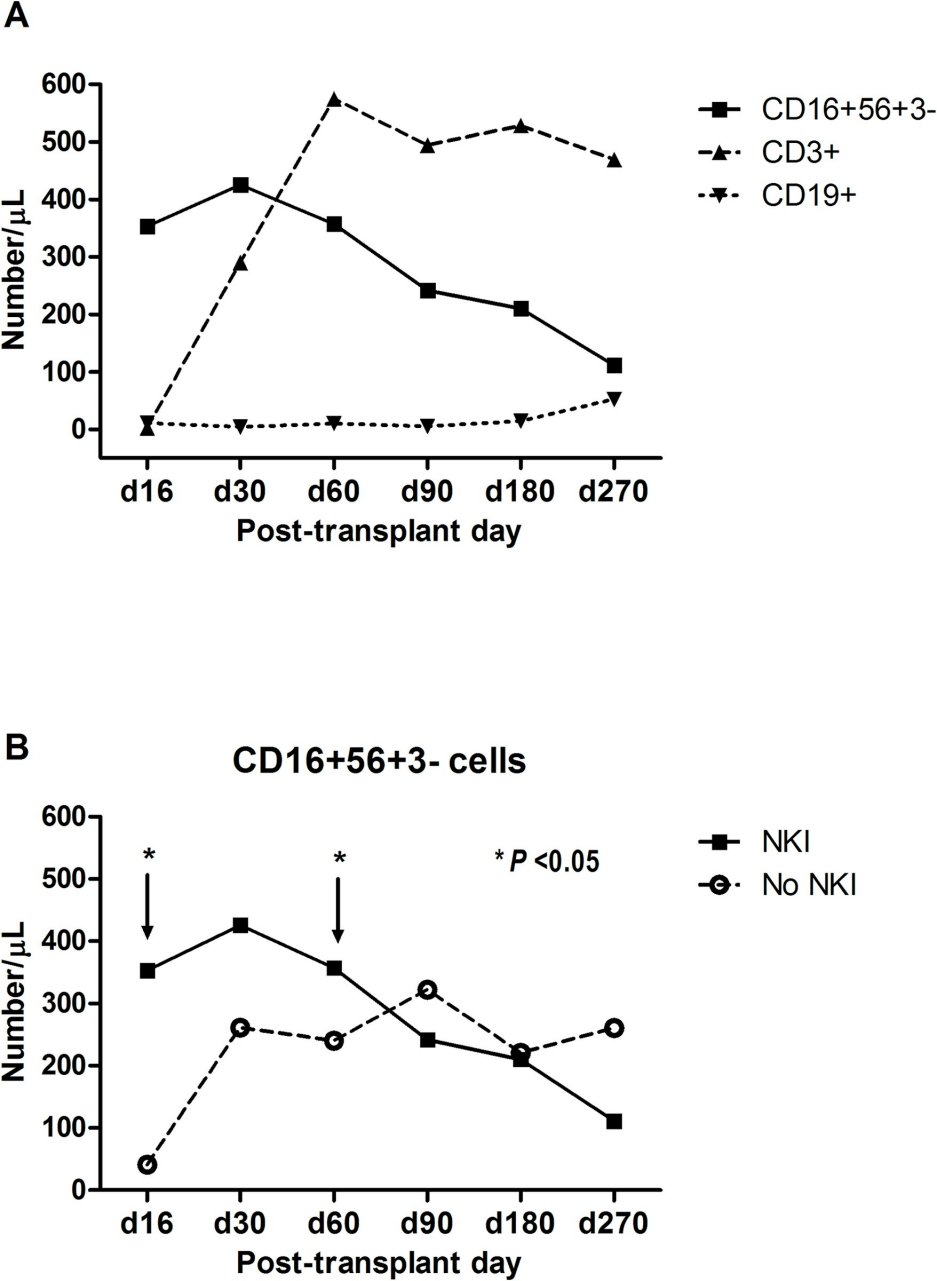
Immune reconstitution after NKI following haplo-SCT. **(**A) Immune reconstitution after NKI following haplo-SCT in six patients who completed three scheduled NKIs. Median values for cell numbers are presented. (B) The number of NK cells was higher until day 60 in the study cohort compared to the reference cohort, who underwent haplo-SCT without NKI.

**Fig 3:**
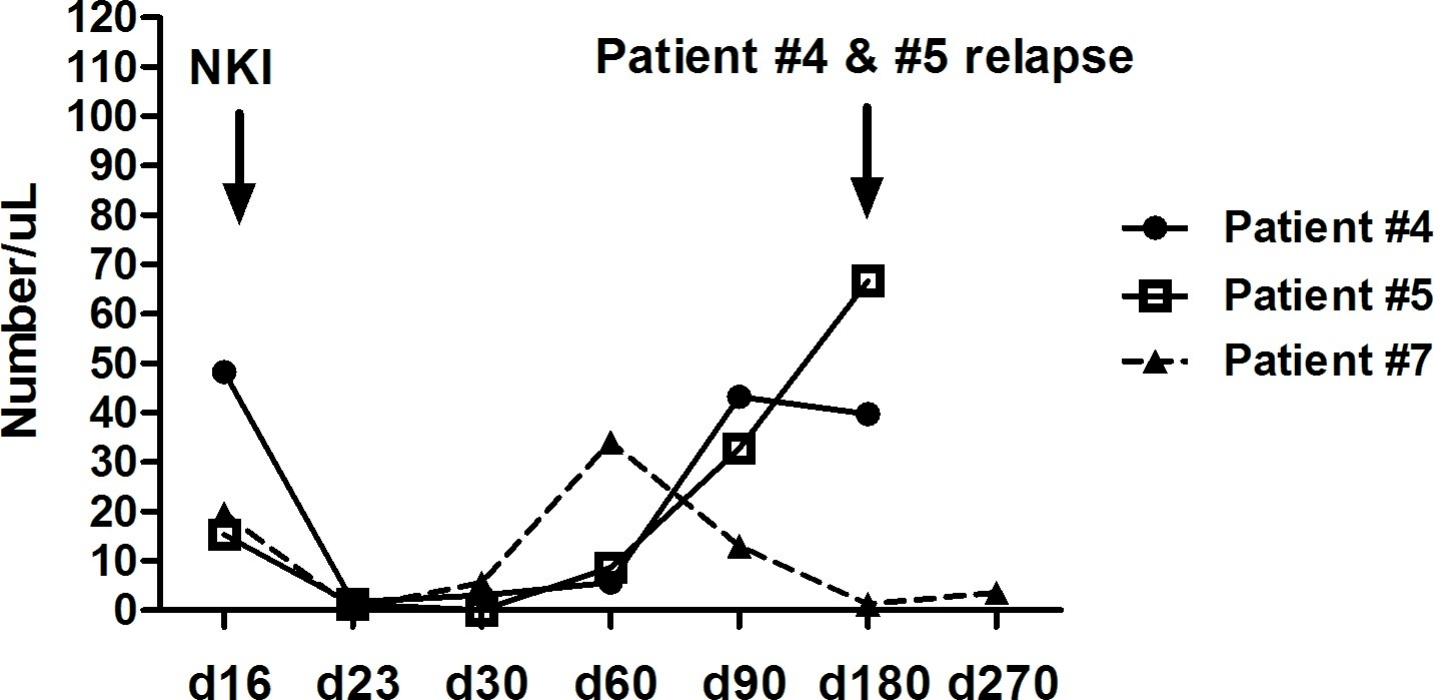
Changes in granulocyte-derived MDSCs after NKI following haplo-SCT. The number of granulocyte-derived MDSCs decreased after NKI. In two patients (patient #3 and #4), the number of granulocyte-derived MDSCs increased from day 90 and tumor relapse/progression had occurred at the six-month tumor evaluation. On the other hand, the number of granulocyte-derived MDSCs did not increase in patient #7, who remained progression-free.

Immune reconstitution was evaluated in six patients who completed three scheduled NKIs. CD16^+^CD56^+^CD3^–^ cells were the predominant lymphocyte population until day 30, CD3^+^ cells were predominant at day 60, and CD19^+^ cells began to increase after day 180 (Fig 2A). When this study’s cohort was compared with the reference cohort, the reconstitution of CD3^+^ cells and CD19^+^ cells was found to be similar (not shown). However, the number of CD16^+^CD56^+^CD3^–^ cells was higher until day 60 in the study cohort (Fig 2B). The number of granulocyte-derived MDSCs decreased after NKI (Fig 3). In two patients (patient #4 and #5), the number of granulocyte-derived MDSCs increased from day 90, and tumor relapse/progression had occurred at the six-month tumor evaluation. On the other hand, the number of granulocyte-derived MDSCs did not increase in patient #7, who remained progression-free.

### Response and survival

**Fig 4:**
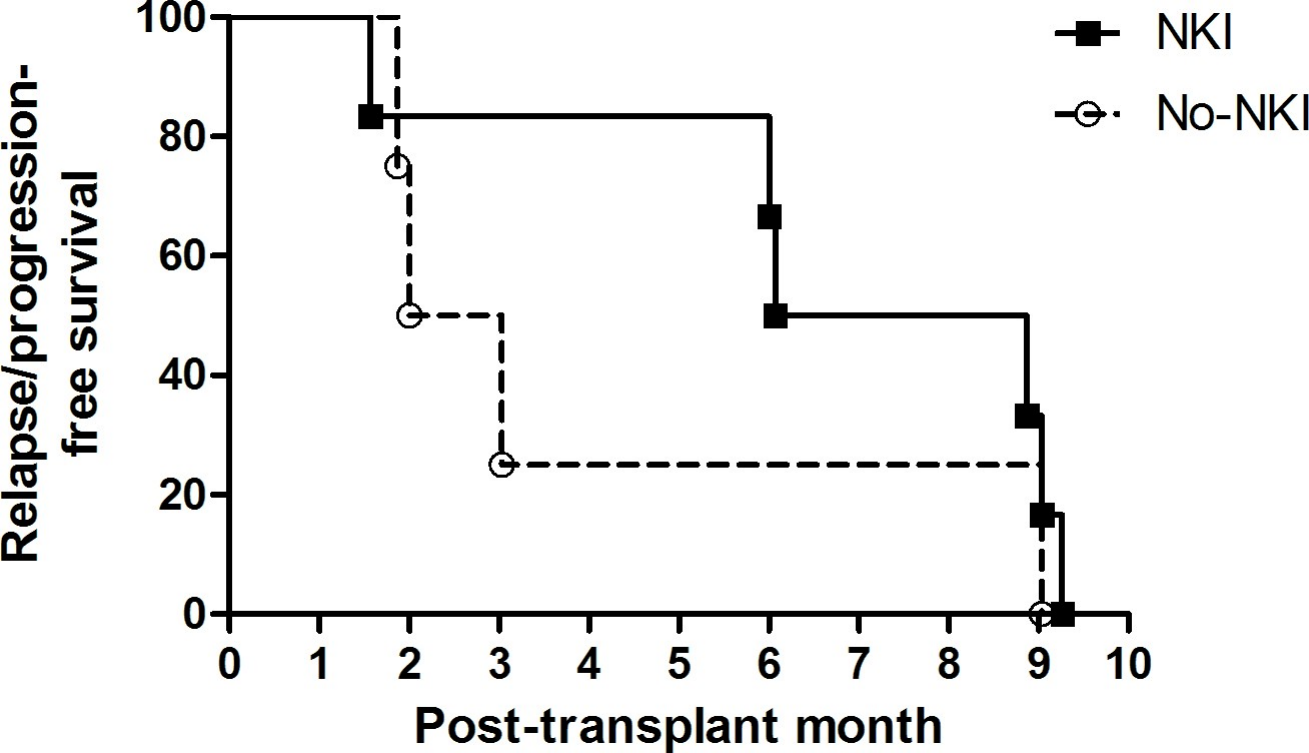
Kaplan-Meier curves in the patients with relapse/progression. The median time to relapse/progression in the current cohort was 7.5 months post-transplant, and that in the reference cohort who underwent haplo-SCT without NKI was 2.5 months post-transplant.

At the three-month tumor evaluation, two patients achieved CR, four patients maintained the same status as at haplo-SCT, and one patient experienced progression (Table 4). During follow-up after NKI following haplo-SCT, a total of six patients experienced relapse/progression at a median of 7.5 (range, 2–9) months post-transplant. Five of them died at a median of 16 (range, 8–29) months post-transplant, and the remaining one remained alive in CR at 45 months post-transplant after salvage treatment including surgery, radiotherapy, and TC chemotherapy. Treatment-related mortality occurred in one patient (patient 7) without tumor progression, as mentioned above. The median time to relapse/progression in the current cohort was 7.5 months post-transplant, which was relatively longer than that in the reference cohort; however there was no statistical difference between the cohorts (*P* = 0.323; Fig 4).

## Discussion

Our previous study suggested that incorporation of HD-MIBG treatment into KIR/HLA-ligand mismatched haplo-SCT might improve outcomes in children with recurrent neuroblastoma [17]. However, in that study, tumor relapse/progression occurred in the early post-transplant period at a median of 2.5 (range, 2–9) months post-transplant. In the current study, under the hypothesis that NKI during the early post-transplant period might prevent early relapse in patients with recurrent neuroblastoma, we evaluated the safety and feasibility of early NKI after haplo-SCT. Our results showed that NKI-related immediate adverse reactions were tolerable, and the incidence of GVHD and infectious complications was similar to those in our previous study [17].

Acute toxicities during NKI were uncommon in previous studies [30–33]. Lee *et al*. reported that most NKI-related acute toxicities were mild except one patient who experienced a grade 2 allergic reaction [34]. Another study reported transient neurologic toxicities such as headache, confusion, delirium, and generalized seizure after NKI; however, those authors reported that these neurologic complications might be related to haplo-SCT toxicity [19]. In the current study, NKI-related acute toxicities were manageable and included fever, chills, and hypertension, and there were no allergic reactions or neurologic complications.

The role of NK cells in the development of GVHD is controversial. Previous studies reported that NK cells had GVT effects without aggravating or inducing GVHD [7,35]. To the contrary, Shah *et al*. reported that *ex vivo*-expanded NK cells may aggravate acute GVHD in T cell-depleted allo-SCT [36]. In the current study, acute GVHD occurred in all patients; however, it was mild to moderate and tolerable. In terms of chronic GVHD, we tapered immune suppression relatively early to enhance GVT if patients could not achieve CR, which may have resulted in the higher incidence of chronic GVHD in our cohort. It is therefore unclear whether NKI increases the incidence of chronic GVHD after haplo-SCT. Further studies are needed to evaluate the association between NKI and development of GVHD.

The number of infused NK cells is an important factor in their persistence after infusion [37]. The optimal doses or times of NKI have not yet been determined. We administered three weekly NKIs with a dose of 3 × 10^7^/kg cells and found that the number of NK cells was higher until day 60 in the study cohort compared to the reference cohort, who underwent haplo-SCT without NKI. Also, it should be noted that the persistence of NK cells was far enhanced as compared with other clinical trials with NKI, in which allogeneic NK cells persisted for 1 to 2 weeks when administered along with immunosuppressive regimens in order to dampen the host T-cell response [33,38,39]. Thus, we suggest that our NKI protocol could maintain a high level of NK cells during the early post-transplant period.

MDSCs can inhibit innate and adaptive immune responses, which may promote tumor angiogenesis, invasion, and metastasis [40]. We found that the number of MDSCs decreased after NKI, like in a previous study in which *ex vivo-*expanded NKI reduced MDSC number [37]. Our data showed that the number of granulocyte-derived MDSCs increased prior to definite tumor progression, consistent with previous studies in which an increased number of MDSCs was associated with tumor progression [41,42]. Overall, our observations that NKI reduced MDSC populations, and enhanced persistency of NK cells suggest that NKI following haplo-SCT could be an effective therapy against cancer.

There are several limitations in this study. First, the number of patients was small. Second, the timing and speed in the tapering of immune suppression were different among patients according to GVHD and tumor status, making the association between NKI and GVHD unclear. Third, although the time to relapse/progression was relatively longer in the study cohort compared to the reference cohort, there was no difference in long-term outcomes between the two cohorts. Therefore, further efforts will be needed to improve long-term outcomes without increasing GVHD, such as the use of anti-GD2 antibody with NKI [43], TCRα/β-depleted [44], CD45RA-depleted grafts [45], or chimeric antigen receptor-modified NK cells [46]. Fourth, failure of stable NK cell production remains a problem. NK cells from universal healthy donors, particularly those who have the KIR BX haplotype or mismatched KIR/HLA-ligand, might be an option in improving transplant outcomes [37].

In summary, our data are supportive of the safety of NKI following haplo-SCT for treating patients with recurrent neuroblastoma. However, the number of patients in our study was too small to draw any definitive conclusions. Therefore, further studies are needed with a larger cohort and new treatment modalities that can improve GVT effects without increasing GVHD to improve outcomes.

## Supporting information

S1 File Clinical trial protocol is available as supporting file. (DOCX)
