# Supplementary material for: Safety and immune cell kinetics after donor natural killer cell infusion following haploidentical stem cell transplantation in children with recurrent neuroblastoma
Source: PLoS One. 2019 Dec 13;14(12):e0225998. doi: 10.1371/journal.pone.0225998 (PMC6910678; doi:10.1371/journal.pone.0225998)
Supplement: S1 File — (DOCX) [file pone.0225998.s001.docx]

| This protocol is for research purposes only and should not be copied, redistributed or used for any other purpose. The procedure of this protocol is intended for use in clinical oncologist in a carefully configured environment and may not be more effective than standard treatment. Responsible investigators associated with this clinical trial should consult with them before using or attaching the procedures in this protocol. |
| --- |

**Safety and immune cell kinetics after donor natural killer cell infusion following haploidentical stem cell transplantation in children with recurrent neuroblastoma**

| **Study chair**  **Ki Woong Sung, M.D. Ph.D.**  Department of Pediatrics, Samsung Medical Center, Sungkyunkwan University School of Medicine, Seoul, Republic of Korea  E-mail: kwsped@skku.edu |
| --- |

**Contents**

[**Treatment scheme** 4](#_Toc16260869)

[**1. Study Description** 5](#_Toc16260870)

[**1-1. Brief Summary** 5](#_Toc16260871)

[**1-2. Detailed Description** 5](#_Toc16260872)

[**2. Outcome Measures** 5](#_Toc16260873)

[**2-1. Primary Outcome Measures** 5](#_Toc16260874)

[**2-2. Other Outcome Measures** 5](#_Toc16260875)

[**3. Background** 6](#_Toc16260876)

[**3-1. Development of treatment for neuroblastoma** 6](#_Toc16260877)

[**3-2. Treatment of recurrent neuroblastoma** 7](#_Toc16260878)

[**3-3. Natural killer cell therapy** 8](#_Toc16260879)

[**3-4. Donor-derived NK cell infusion** 8](#_Toc16260880)

[**3-5. The objectives of this study** 9](#_Toc16260881)

[**4. Study enrollment and patient eligibility** 10](#_Toc16260882)

[**4-1. Inclusion Criteria:** 10](#_Toc16260883)

[**4-2. Exclusion Criteria:** 10](#_Toc16260884)

[**5. Treatment plan** 10](#_Toc16260885)

[**5-1. Treatment prior to haplo-SCT** 10](#_Toc16260886)

[**5-2. Donor selection** 11](#_Toc16260887)

[**5-3. NK cell generation and stem cell collection** 12](#_Toc16260888)

[**5-4. Conditioning** 12](#_Toc16260889)

[**5-5. NKI** 13](#_Toc16260890)

[**5-6. GVHD prophylaxis and treatment** 13](#_Toc16260891)

[**5-7. Infection surveillance and prophylaxis** 14](#_Toc16260892)

[**5-8. Chimerism study and immune monitoring** 14](#_Toc16260893)

[**5-9. Toxicity and response assessment** 14](#_Toc16260894)

[**6. Adverse events** 16](#_Toc16260895)

[**6-1. Adverse events associated with haplo-SCT** 16](#_Toc16260896)

[**6-2. NKI** 16](#_Toc16260897)

[**6-3. IL-2** 17](#_Toc16260898)

[**7. More Information** 18](#_Toc16260899)

# **
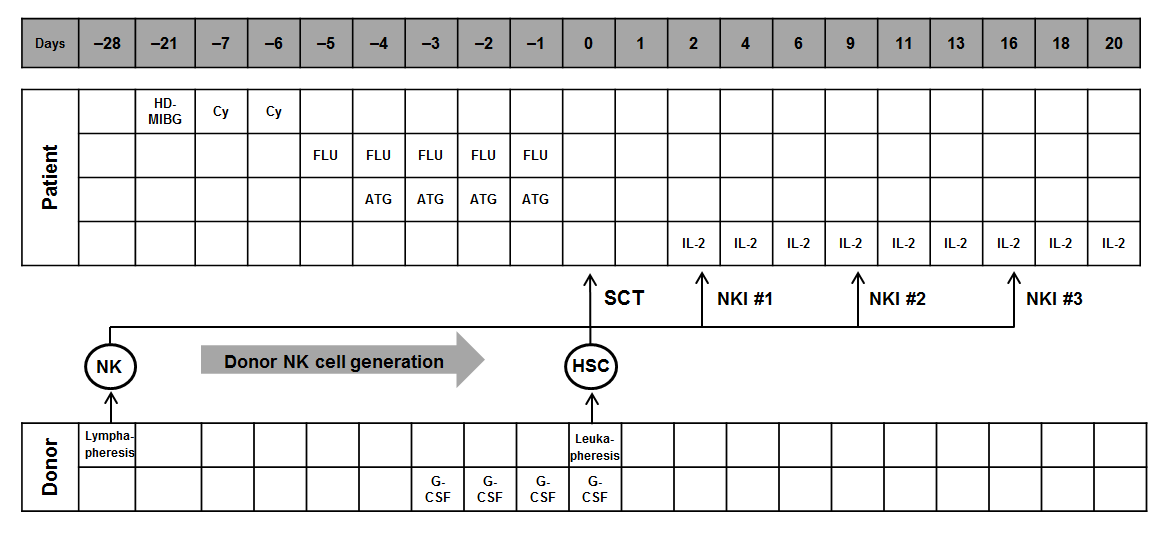
Treatment scheme**

Abbreviation: ATG = anti-thymocyte globulin; Cy = cyclophosphamide; FLU = fludarabine; HD-MIBG = high-dose ^131^I-metaiodobenzylguanidine treatment; HSC = hematopoietic stem cells; IL-2 = interleukin-2; NK = natural killer cell; NKI = NK infusion; SCT = stem cell transplantation.

# **1. Study Description**

## **1-1. Brief Summary**

To evaluate feasibility natural killer (NK) cell infusion after haploidentical stem cell transplantation in patients with neuroblastoma who failed after tandem high-dose chemotherapy and autologous stem cell transplantation.

## **1-2. Detailed Description**

Haploidentical (Haplo-SCT) following reduced-intensity conditioning (RIC) regimen will be performed in patients with recurrent/relapsed neuroblastoma. Both parents will be evaluated for their killer cell immunoglobulin-like receptor (KIR) genotype and phenotype and the one with the greatest degree of KIR-ligand mismatch with the patient will be selected as the donor. In addition, ex-vivo expanded NK cells derived from the donor will then be administered after haplo-SCT to increase the graft-versus-tumor (GVT) effect. Low-dose IL-2 will be given after expanded NK cell infusion to enhance NK cell alloreactivity.

# **2. Outcome Measures**

## **2-1. Primary Outcome Measures**

Number of study patients with adverse events as a measure of safety of haploidentical stem cell transplantation and NK cell infusion

## **2-2. Other Outcome Measures**

- Number of patients developing graft-versus-host disease (GVHD)
- Infectious complications
- Chimerism and immune monitoring
- Treatment outcomes

# **3. Background**

## **3-1. Development of treatment for neuroblastoma**

Neuroblastoma, a neoplasm of the sympathetic nervous system, is the second most common solid tumor in children, constituting 6–10% of all childhood malignancies. This disease is a heterogeneous malignancy with prognoses ranging from spontaneous regression to a high risk of progression and death, depending on various clinical and biological factors. The most important prognostic factors are the age at diagnosis, stage of disease, histology, *MYCN* amplification status, and DNA ploidy. Patients with neuroblastoma are classified into low-, intermediate-, and high-risk groups based on their prognostic factors, and treatments stratified by risk classification show improved survival outcomes. Treatment outcomes were excellent for patients who had low- and intermediate-risk neuroblastoma with a conventional treatment modality; however, the prognosis of high-risk neuroblastoma patients is generally poor with conventional treatment alone. The development of high-dose chemotherapy and autologous stem cell transplantation (HDCT/auto-SCT) has improved the treatment outcomes of patients with high-risk neuroblastoma in recent decades. However, the 5-year event-free survival and overall survival rates from randomized trials by the Children’s Oncology Group were 30% and 39%, respectively, which remains disappointing. Recently, several researchers have shown that tandem HDCT/auto-SCT may be a feasible strategy to improve the outcomes; however, approximately half of patients experienced tumor relapse, even after tandem HDCT/auto-SCT. Since conventional chemotherapy was found to be ineffective in patients with recurrent neuroblastoma, and they were also vulnerable to additional intensive treatments, there was no curative option for these patients until recently.

## **3-2. Treatment of recurrent neuroblastoma**

Allogeneic SCT (allo-SCT) has been used for the treatment of hematologic malignancies and high-risk or refractory/recurrent solid tumors. The response of allo-SCT by solid tumors has shown an association with GVHD, and previous results suggested that a GVT effect might be a treatment option for patients with recurrent solid tumors. This GVT effect has been demonstrated in patients with refractory neuroblastoma who were receiving allo-SCT. However, the treatment-related complications caused by allo-SCT were very high in patients who experienced relapse after HDCT/auto-SCT, which was associated with intensive conditioning regimens in standard allo-SCT. For these reasons, reduced-intensity conditioning (RIC) regimens have been developed that may induce a GVT effect without the resulting treatment-related morbidities for patients at a high risk of regimen-related mortality, particularly patients who previously received tandem HDCT/auto-SCT treatment. Recently, haplo-SCT has been adapted for the treatment of solid tumors; it has also shown feasibility and acceptable toxicities in children with recurrent solid tumors including neuroblastoma. To enhance the anti-tumor effect of patients with recurrent neuroblastoma receiving haplo-SCT, high-dose ^131^I-metaiodobenzylguanidine (HD-MIBG) treatment has also been performed, which showed tolerable toxicities and potential anti-tumor effects.

## **3-3. Natural killer cell therapy**

In haplo-SCT, in which T cells are usually depleted to prevent unacceptable GVHD, donor natural killer (NK) cells may play an important role in eliminating residual tumor cells. NK cells are innate effector lymphocytes and show cytotoxicity against tumor cells until T cell recovery with decreased expression of major histocompatibility class I antigens. The activity of NK cells is controlled by a network of stimulatory activating and inhibitory receptors. Previous studies have shown that the selection of donors with killer cell immunoglobulin-like receptors (KIR) mismatched with recipient human leukocyte antigen (HLA) or group B KIR haplotypes improved the transplant outcomes in several malignancies. Previously, a pilot study incorporating HD-MIBG treatment into KIR/HLA-ligand mismatched haplo-SCT for children with recurrent neuroblastoma was performed. Seven patients were enrolled in this previous study, of which four died of tumor relapse/progression, one died of treatment-related mortality, and the remaining two survived without disease recurrence. This previous study showed that tumor relapse/progression occurred during the early post-transplant period, (median, 2.5 months; range, 2–9 months), suggesting the need for further effective treatments to prevent early relapse after haplo-SCT.

## **3-4. Donor-derived NK cell infusion**

The chimeric status of recipients after haplo-SCT provides a platform for immunotherapy using donor-derived NK cells. This immunotherapy, known as donor-derived NK cell infusion (NKI), has developed into an effective treatment for recurrent malignancies. Since the main risk of donor-derived NKI is the aggravation of GVHD, which can be a fatal complication, adapted treatments such as T-cell depleted donor-derived NKI have been developed to prevent GVHD. Several clinical trials have been performed to determine whether purified donor NKI can facilitate engraftment and induce GVT effects in haplo-SCT; these investigations have demonstrated its feasibility and efficiency.

Previously, Green Cross (GC) LabCell Corp. (Yongin, South Korea) successfully expanded NK cells acquired from healthy donors under good manufacturing practice (GMP) conditions, and showed that they were effective in preventing tumor progression in severe combined immunodeficient mice injected with human lymphoma cells. In a subsequent study, GC LabCell reported a phase I study of NKI in patients with malignant lymphoma or recurrent solid tumors, and showed that the maximum dose (3 × 10^7^ cells/kg, triple infusion) of NKI was acceptable without significant adverse events.

A previous study reported that neuroblastoma cells display decreased class I HLA expression, which suggests that NKI may be effective in killing neuroblastoma cells. It was therefore hypothesized that donor-derived NKI following haplo-SCT could be an effective treatment option for recurrent neuroblastoma, and planned a pilot study investigating the safety and feasibility of NKI produced by GC LabCell following haplo-SCT in children with recurrent neuroblastoma.

## **3-5. The objectives of this study**

Clinical trials exploring the feasibility of NKI after haplo-SCT have previously been performed in patients with several malignancies. However, studies in children with neuroblastoma have been limited. It was, therefore, hypothesized that early donor-derived NKI following haplo-SCT may be helpful in preventing early relapse and improving the survival of pediatric recurrent neuroblastoma patients. Thus, a pilot study was performed to explore the safety and immune cell kinetics of donor-derived NKI following haplo-SCT in children with recurrent neuroblastoma who were previously unsuccessfully treated with tandem HDCT/auto-SCT.

# **4. Study enrollment and patient eligibility**

## **4-1. Inclusion Criteria:**

- Age < 21 years old
- Patients with high-risk solid tumors who failed prior HDCT/auto-SCT
- Patients with a suitable haploidentical donor
- Recurrent/relapsed neuroblastoma
- Stable disease with salvage chemotherapy after relapse
- Patients without active infectious disease

## **4-2. Exclusion Criteria:**

- Organ dysfunction (NCI common toxicity criteria grade > 2)
- Progression of disease despite salvage chemotherapy

# **5. Treatment plan**

## **5-1. Treatment prior to haplo-SCT**

Salvage chemotherapy will be administered in order to reduce the tumor burden as much as possible prior to haplo-SCT. An ICE (ifosfamide + carboplatin + etoposide) regimen will be used for first-line salvage treatment, and a TC (topotecan + cyclophosphamide) regimen will be used for second-line salvage chemotherapy in patients with severe bone marrow suppression or refractory response with the first-line regimen (Table 1 and 2). The duration of salvage chemotherapy prior to haplo-SCT depended on tumor response and patient tolerance. Tumors will be surgically resected whenever possible. Local radiotherapy will be also delivered to recurrent or metastatic sites whenever possible.

**Table 1.** ICE chemotherapy

| *Regimen* | *Drug* | *Dose* | *Schedule* | *Total dose* |
| --- | --- | --- | --- | --- |
|  | Ifosfamide | 1200 mg/m^2^/day | Days 0−4 | 6,000 mg/m^2^ |
|  | Carboplatin | 400 mg/m^2^/day | Days 0 & 1 | 800 mg/m^2^ |
|  | Etoposide | 100 mg/m^2^/day | Days 0−4 | 500 mg/m^2^ |

**Table 2.** TC chemotherapy

| *Regimen* | *Drug* | *Dose* | 0 | 1 | 2 | 3 | 4 |
| --- | --- | --- | --- | --- | --- | --- | --- |
|  | Topotecan | 0.75 mg/m^2^/day | 1 | 1 | 1 | 1 | 1 |
|  | Cyclophosphamide | 250 mg/m^2^/day | 1 | 1 | 1 | 1 | 1 |

## **5-2. Donor selection**

Typing of HLA A, B, C, DRB1, and DQB1 will be performed using high-resolution PCR sequence-based typing, and KIR genotyping will be performed from donor DNA samples using a PCR-based sequence-specific oligonucleotide technique. A KIR/HLA-ligand mismatch will be defined by incompatibility between the inhibitory donor KIR and recipient HLA class I alleles. Donor KIR haplotypes will be categorized as AA (homozygous for group A KIR haplotypes) or BX [either one (A/B heterozygotes) or two (B/B homozygotes) group B haplotypes]. The KIR B haplotype-defining loci will be *KIR2DL5, 2DS1, 2DS2, 2DS3, 2DS5,* or *3DS1*. Genotypes will be also assigned for the centromeric and telomeric regions of the KIR locus. A haploidentical parent donor with KIR/HLA-ligand mismatch and/or KIR BX haplotype will be preferred.

## **5-3. NK cell generation and stem cell collection**

For NK cell production, haploidentical parent donors will undergo lymphapheresis on day -28, and CD3^+^ cell–depleted peripheral blood mononuclear cells (PBMCs) will be frozen at -196°C. Peripheral blood mononuclear cells will be thawed (days -12, -5, and 2) 14 days before each of the three planned infusions (days 2, 9, and 16) to allow each preparation and infusion of fresh cells. The thawed PBMCs will expand under good manufacturing practice conditions in GC LabCell. CD3^+^ cell–depleted PBMCs will be expanded at a seeding concentration of 2 × 10^5^ cells/mL in CellGro SCGM serum-free medium (CellGenix, Germany) with 1% autologous plasma, 1 × 10^6^ cells/mL irradiated (2,000 rad) autologous PBMCs, 10 ng/mL anti-CD3 monoclonal antibody (Orthoclon, Switzerland), and 500 IU/mL of interleukin-2 (IL-2; Proleukin, Switzerland) in an A-350N culture bag (NIPRO, Japan). NK cells will be fed fresh medium with 500 IU/mL of IL-2 every 2 days until they will be harvested after 14 days. The cytotoxicity of *ex-vivo* expanded donor NK cells will be measured using K562, SK-N-SH, and NB-1691 cells by calcein releasing assay. For peripheral blood stem cell (PBSC) collection, haploidentical parent donors will receive 5­–10 μg/kg of G-CSF subcutaneously once daily for four days; PBSCs will be collected and transplanted without manipulation on day 0.

## **5-4. Conditioning**

At 21 days prior to transplant, all children will receive a single 1-hour intravenous infusion of ^131^I-MIBG (18 mCi/kg) with potassium iodide for thyroid protection and intravenous hydration. A cyclophosphamide + fludarabine + rabbit anti-thymocyte globulin regimen will be used for reduced-intensity conditioning (Table 3).

**Table 3. Reduced-intensity conditioning regimen**

| *Drug* | *Dose* | *-21* | *…* | *-7* | *-6* | *-5* | *-4* | *-3* | *-2* | *-1* | *0* |
| --- | --- | --- | --- | --- | --- | --- | --- | --- | --- | --- | --- |
| ^131^I-MIBG | 18 mCi/kg | 1 |  |  |  |  |  |  |  |  |  |
| Cyclophosphamide | 60 mg/kg/day |  |  | 1 | 1 |  |  |  |  |  |  |
| Fludarabine | 30 mg/m^2^/day |  |  |  |  | 1 | 1 | 1 | 1 | 1 |  |
| ATG | 2.5 mg/kg/day |  |  |  |  |  | 1 | 1 | 1 | 1 |  |

## **5-5. NKI**

Patients received 3 × 10^7^/kg of *ex-vivo* will expand donor NK cells on days 2, 9, and 16 post-transplant. Donor NK cells will be infused over 1 hour through a central venous catheter after pheniramine pre-treatment. Patients will receive IL-2 (1 × 10^6^ IU/m^2^/day) subcutaneously to activate infused donor NK cells on days 2, 4, 6, 9, 11, 13, 16, 18, and 20. On the day of NKI, IL-2 will be administered after a 4-hour observation period post-NKI.

## **5-6. GVHD prophylaxis and treatment**

Cyclosporine (CSA) and short-course methotrexate will be used to prevent GVHD. CSA will be administered from day -1 at a dose adjusted to maintain blood concentration in the range of 150–300 ng/mL. Methotrexate will be administered at a dose of 15 mg/m^2^ on day 1 and at 10 mg/m^2^ on days 3 and 6, followed by folic acid rescue. The timing and speed of CSA tapering will be determined by GVHD and tumor status of each patient. If the patient does not achieve complete response (CR), early tapering of CSA will be considered to enhance GVT. If acute GVHD develops during CSA prophylaxis or tapering, the CSA dose will be increased. If ≥ grade II acute GVHD continues despite an increase in CSA dose, methylprednisolone (1–2 mg/kg/day) will be added with subsequent tapering in responsive cases. In refractory GVHD, mycophenolate mofetil will be added to reduce use of steroid. Acute and chronic GVHD will be assigned grades and stages based on previously described standard clinical criteria.

## **5-7. Infection surveillance and prophylaxis**

Antifungal prophylaxis will be administered until hospital discharge or during steroid treatment. Acyclovir will be used to prevent viral reactivation by day 30, and trimethoprim-sulfamethoxazole will be used from engraftment to day 180 or until immunosuppressant discontinuation. Cytomegalovirus (CMV), Epstein-Barr virus (EBV), and BK virus (BKV) surveillance will be performed weekly during the first three months post-transplant and then monthly thereafter if no viral reactivation occurred. If CMV or EBV load will be increasing, ganciclovir or rituximab will be started as preemptive therapy, respectively.

## **5-8. Chimerism study and immune monitoring**

Donor/recipient chimerism will be evaluated at 30, 60, 90, and 180 days post-transplant in peripheral blood. Immunologic recovery will be assessed by immunophenotyping of PBMCs (CD3^+^, CD19^+^, and CD16^+^CD56^+^CD3^–^ cells) from recipients at 16, 30, 60, 90, 180, and 270 days post-transplant. Granulocyte-derived myeloid-derived suppressor cells (MDSCs) by lymphogating of Lin^–^CD14^–^HLA-DR^–^CD11b^+^CD33^+^CD15^+^ cells will be analyzed to identify the association between the levels of these immune cells and relapse/progression.

## **5-9. Toxicity and response assessment**

NKI-related immediate adverse reactions will be defined as adverse reactions that developed from initiation of NKI to 4 hours after completion of NKI. Toxicity will be recorded according to the common toxicity criteria (version 4.0) outlined by the US National Cancer Institute. Tumor response evaluation will be performed prior to HD-MIBG treatment and every three months for the first year post-transplant. International response criteria for neuroblastoma will be used to evaluate treatment response.

International Neuroblastoma Response Criteria (INRC)

- CR: no identifiable tumor with normal catecholamine
- VGPR (very good partial response): a reduction of the primary tumor by 90–99% with normal catecholamine with or without any residual 99Tc bone changes
- PR: reduction of the primary tumor and metastatic tumor by >50%.
- MR (mixed response): reduction of any measurable lesion by >50% with a reduction of any other lesion by <50%
- PD (progressive disease):any new lesion or increase of any measurable lesion by >25%

Tumor response evaluation

- CT/MRI, FDG/PET scan, bone scan, MIBG scan
- Baseline, 3 mo, 6 mo, 9 mo, 12 mo, 16 mo, 20 mo, 24 mo, 30 mo, 36 mo

Organ function test

- Renal: BUN/Cr, glucose, cystatin-C, 24hr urine (protein, Ccr, urine volume), Random urine (Prot/Alb, Ca, P, Uric acid, Creatinine), ß2-MG, ß – NAG
- Pulmonary : Peak flow meter or PFT, non-contrast Chest CT, lung perfusion scan, Diffusion capacity
- Cardiology: 2D-echocardiography, EKG
- Audiometry
- Thyroid function test

# **6. Adverse events**

## **6-1. Adverse events associated with haplo-SCT**

- Fever (>90%)
- Veno-occlusive disease (15-20%)
- CMV reactivation (80-90%)
- GVHD (30-50%)
- Post-transplant lymphoproliferative disease (10%)

## **6-2. NKI**

- Anaphylaxis or hypersensitivity
- Asthenia
- Chills
- Fatigue
- Fever
- Headache
- Hypertension
- Hypotension
- Myalgia
- Nervous system disorders
- Skin rash
- Sweating

## **6-3. IL-2**

1) >10%:

- Cardiovascular: Hypotension (71%; grade 4: 3%), peripheral edema (28%), tachycardia (23%), edema (15%), vasodilation (13%), supraventricular tachycardia (12%; grade 4: 1%), cardiovascular disorder (11%; includes blood pressure changes, HF and ECG changes)
- Central nervous system: Chills (52%), confusion (34%; grade 4: 1%), fever (29%; grade 4: 1%), malaise (27%), somnolence (22%), anxiety (12%), pain (12%), dizziness (11%)
- Dermatologic: Rash (42%), pruritus (24%), exfoliative dermatitis (18%)
- Endocrine & metabolic: Acidosis (12%; grade 4: 1%), hypomagnesemia (12%), hypocalcemia (11%)
- Gastrointestinal: Diarrhea (67%; grade 4: 2%), vomiting (19% to 50%; grade 4: 1%), nausea (19% to 35%), stomatitis (22%), anorexia (20%), weight gain (16%), abdominal pain (11%)
- Hematologic: Thrombocytopenia (37%; grade 4: 1%), anemia (29%), leukopenia (16%)
- Hepatic: Hyperbilirubinemia (40%; grade 4: 2%), AST increased (23%; grade 4: 1%)
- Neuromuscular & skeletal: Weakness (23%)
- Renal: Oliguria (63%; grade 4: 6%), creatinine increased (33%; grade 4: 1%)
- Respiratory: Dyspnea (43%; grade 4: 1%), lung disorder (24%; includes pulmonary congestion, rales, and rhonchi), cough (11%), respiratory disorder (11%; includes acute respiratory distress syndrome, infiltrates and pulmonary changes)
- Miscellaneous: Antibody formation (66% to 74%), infection (13%; grade 4: 1%)

2) 1% to 10%:

- Cardiovascular: Arrhythmia (10%), cardiac arrest (grade 4: 1%), MI (grade 4: 1%), ventricular tachycardia (grade 4: 1%)
- Central nervous system: Coma (grade 4: 2%), stupor (grade 4: 1%), psychosis (grade 4: 1%)
- Gastrointestinal: Abdomen enlarged (10%)
- Hematologic: Coagulation disorder (grade 4: 1%; includes intravascular coagulopathy)
- Hepatic: Alkaline phosphatase increased (10%)
- Renal: Anuria (grade 4: 5%), acute renal failure (grade 4: 1%)
- Respiratory: Rhinitis (10%), apnea (grade 4: 1%)
- Miscellaneous: Sepsis (grade 4: 1%)

# **7. More Information**

| Responsible Party: | Ki Woong Sung, Professor, Samsung Medical Center |
| --- | --- |
| ClinicalTrials.gov Identifier: | [NCT01807468](https://clinicaltrials.gov/show/NCT01807468) |
| Other Study ID Numbers: | CR0113061 |
